# Systematic monitoring identified a high incidence of hypopituitarism following combined ipilimumab plus nivolumab therapy for metastatic melanoma

**DOI:** 10.3389/fendo.2026.1827644

**Published:** 2026-05-08

**Authors:** Mahir Hasan, Wolfram Samlowski, Samer Nakhle

**Affiliations:** 1 Touro University College of Osteopathic Medicine, Las Vegas, NV, United States; 2 Department of Internal Medicine, University of Nevada, Las Vegas (UNLV), Las Vegas, NV, United States; 3 Nevada Oncology Specialists, Las Vegas, NV, United States; 4 Palm Medical Group, Las Vegas, NV, United States

**Keywords:** cancer immunotherapy, checkpoint inhibitors, endocrinopathy, hypophysitis, hypothyroidism, progression-free survival

## Abstract

**Introduction:**

Combination immunotherapy with ipilimumab and nivolumab has improved long-term progression-free and overall survival in patients with metastatic melanoma. Unfortunately, a high percentage of patients develop immune-related side effects, including endocrinopathy. The incidence and timing of hypopituitarism remains poorly characterized.

**Methods:**

We routinely performed laboratory screening for hypopituitarism and hypothyroidism every 3–4 weeks on each treatment visit for patients receiving first-line ipilimumab plus nivolumab therapy for metastatic melanoma. A retrospective analysis of patient treatment records was performed to determine the incidence and timing of biochemical evidence for endocrinopathy.

**Results:**

Records from 59 sequential patients treated with first-line combination immunotherapy were reviewed. Endocrinopathy was detected in 44.1%, including 18.7% with hypopituitarism and 25.4% with primary hypothyroidism. The median time to onset of was 72.5 ± 107.3 and 58 ± 48.7 days, respectively. Only one patient developed delayed onset of hypothyroidism, 3 months after elective treatment discontinuation. Development of endocrinopathy trended with improved clinical outcomes. All patients underwent prompt endocrine replacement therapy, and only one patient required hospitalization for symptomatic hypopituitarism.

**Conclusions:**

Both the standard and alternate regimens of ipilimumab plus nivolumab treatment resulted in biochemical evidence for endocrinopathy in 54.6% and 38.9% of treated patients, respectively. Endocrinopathy always presented during or shortly following active treatment. Delayed onset of endocrinopathy was uncommon. Prompt replacement therapy allowed continuation of immune checkpoint inhibitors and minimized the risk for hospitalizations. Due to the high incidence of hypopituitarism, routine monitoring of both pituitary and thyroid and function of patients is strongly recommended receiving combination immunotherapy.

## Introduction

Melanoma currently represents the fifth most common cancer in men and women in the United States. In 2025, it is estimated that there will be over 104,000 patients who develop invasive melanomas, resulting in an estimated 8430 deaths from metastatic disease (1).

In the past, metastatic melanoma was a far more devastating illness. In the chemotherapy era, metastatic melanoma patients had a median survival of only 5.9 to 6.5 months, with a progress-free survival (PFS) range of 1.6 to 1.8 months (2–5). In a meta-analysis of clinical trials from this era, median progression-free survival was 15% at 6 months, with only 25% of patients remaining alive at 1 year (4).

Fortunately, since 2013 there has been a steep decline in mortality of metastatic melanoma (6.1% decrease per year) (1), attributable to the development of effective targeted therapy and immunotherapy (6, 7). Treatment of BRAF-V600 locus mutation-expressing melanomas (about 40% of melanoma patients) with combined BRAF/MEK inhibitor therapy resulted in a 5-year survival rate of approximately 34% (8–10). However, progression-free survival was only 19% at 5 years, despite continuation of oral targeted therapy (8–10).

The development of immune checkpoint inhibitors (ICI) has led to a further improvements in the survival in metastatic melanoma patients (11). Monotherapy with ipilimumab, a CTLA-4 specific antibody, resulted a median progression-free survival of 2.9 months, with an overall survival (OS) of 19.9 months. Notably, this agent produced a small percentage of long-term remissions, and a five-year survival rate of 26% (12). Treatment with PD-1 directed monoclonal antibodies further improved clinical outcomes, compared to ipilimumab. Treatment with nivolumab monotherapy improved median PFS to 6.9 months with a median OS of 36.9 months, and a five-year survival of 44% (12). Treatment with another PD-1 antibody, pembrolizumab, resulted in a median PFS of 9.4 months, with a median OS of 32.7 months, and a 5-yr survival of 39.9% (13). Subsequently a combination of CTLA4 and PD1-directed monoclonal antibodies was tested and proved even more effective. Treatment with ipilimumab plus nivolumab resulted in a median PFS of 11.5 months, a median OS of 71.9 months, and a five-year survival rate of 52% (12). Many of responding patients achieved a durable remission, allowing treatment discontinuation.

Thus, combination immunotherapy has markedly improved treatment outcomes for metastatic melanoma, resulting in an increasing number of patients who can achieve a durable complete remission. Unfortunately, these advances have come at a significant price. All ICI therapy agents are associated with a unique spectrum of side effects that result from excessive activation of the immune system, termed immune-related adverse events (irAE). The frequency of irAE is markedly increased during dual checkpoint blockade with ipilimumab plus nivolumab (14). Virtually any organ system can become involved (15, 16). The most common irAE include fatigue, rashes, colitis, immune hepatitis, pneumonitis, and endocrinopathy (17). Amongst these side effects, endocrinopathy is relatively unique, as it appears to have an early onset, but represents one of the few toxicities that is likely to persist long-term (18).

There have been varying estimates of the incidence of endocrinopathies, such as hypothyroidism and hypopituitarism following checkpoint inhibitor therapy (15, 19, 20). This is in part, as many publications describe pooled populations of patients receiving ICI monotherapy, as well as combination therapy. Endocrinopathy was frequently only detected only after patients developed symptoms.

We have employed routine endocrine monitoring in all ICI-treated patients in our clinic. We therefore performed a retrospective review of the incidence and timing of biochemical evidence for endocrinopathy in metastatic melanoma patients who were treated with first-line ipilimumab plus nivolumab. We also evaluated the impact of endocrinopathy on eventual treatment outcomes, such as progression-free (PFS) and overall survival (OS).

## Materials and methods

### Patient identification

Potential patients for this retrospective data analysis were identified by searching a HIPAA-compliant iKnowMed clinical database (McKesson, Woodlands, TX). All patients with metastatic melanoma who received first line treatment with ipilimumab and nivolumab for cutaneous melanoma by a single physician (W.S.) were identified. Patients with non-cutaneous melanoma (e.g., uveal or mucosal melanoma) were excluded. Patients who never received ICI treatment or received only one dose were also excluded from this analysis.

### Data extraction

Patient records were individually accessed, and relevant data was extracted into password protected Excel Sheet (version 16.94, Microsoft Corporation, Redmond, WA). Each patient was assigned a unique patient identifier. Demographic data recorded included age, gender, date of birth (DOB), and race. Additional patient information extracted included potential driver mutation status and pretreatment lactate dehydrogenase (LDH) levels. In addition, whether patients were still receiving immunotherapy at the time of endocrinopathy diagnosis was recorded, to determine if delayed onset occurred following elective treatment discontinuation. Furthermore, we recorded the type of ICI treatment patient received along with ICI start date, ICI end date, specific ICI regimen and the number of treatment doses administered. Progression-free rate and overall survival rate was calculated from initial ICI start date. We documented all other ICI-induced toxicities. After the completion of data collection from iKnowMed into the study’s spreadsheet, all patient identifying information was deleted prior to analysis. This study design was reviewed by the Western (WGC) Institutional Review Board (IRB) chair and was deemed exempt from a full IRB review.

### Identification of endocrine abnormalities

The presence of endocrinopathy prior to treatment was recorded, as was the date of onset of abnormal thyroid or pituitary function tests related to the treatment start date. Patients were routinely monitored for development of endocrinopathy prior to each treatment (every 3–4 weeks). This included laboratory testing for thyroid stimulating hormone (TSH), free tetraiodothyronine (FT4), adrenocorticotropic hormone (ACTH) and random cortisol. Primary hypothyroidism was identified based on elevated TSH and low FT4. Transient elevations in FT4 and suppression of TSH was felt to indicate hyperthyroidism induced by autoimmune thyroiditis. Hypopituitarism was identified based on decreased ACTH, cortisol, TSH and FT4. Primary adrenal insufficiency was expected to produce an elevated ACTH plus a low serum cortisol (corrected for diurnal variation).

Medication records were also reviewed to identify the use of systemic corticosteroids or other agents that could potentially suppress the hypothalamic-pituitary-adrenal axis and confound the interpretation of cortisol measurements. None of our patients were receiving glucocorticosteroids at baseline. Borderline cortisol or ACTH results were interpreted in relation to sequential laboratory testing and development of clinical symptoms (e.g., fatigue, decreased blood pressure, orthostatic symptoms) to indicate the need for endocrine replacement therapy. Routine endocrine monitoring in this retrospective analysis focused primarily on thyroid and adrenal axis testing (TSH, FT4, ACTH, and cortisol), while additional pituitary hormones (LH/FSH, prolactin, GH/IGF-1) were not systemically measured.

### Treatment regimens

All patients were treated with one of two ipilimumab/nivolumab regimens. These consisted of either 3 mg/kg ipilimumab plus 1 mg/kg nivolumab i.v. every 3 weeks (standard regimen) or 1 mg/kg ipilimumab plus 3 mg/kg nivolumab i.v. every 3 weeks (alternate or “flipped” regimen) (21). Responding patients were continued on nivolumab maintenance (480 mg i.v. monthly). Patients who achieved a confirmed complete remission based on radiographs or biopsies of residual lesions, underwent elective discontinuation as previously described (22).

### Response assessment

Clinical responses of metastatic melanoma patients was evaluated as the best objective response (BORR) at 12 months (23), based on RECIST 1.1 criteria (24). A complete response (CR) was defined as disappearance of all target lesions. A partial response (PR) was defined as at least a 30% decrease in the sum of diameters of target lesions, compared to baseline measurements. Progressive Disease (PD) was defined by at least a 20% increase in the sum of diameters of target lesions, compared to baseline. Stable disease (SD) did not meet the criteria for CR, PR or PD.

### Statistical assessment

Basic statistical analysis, such as mean, median, standard deviation were calculated via Excel spreadsheet. We analyzed overall survival (OS) and progression free survival (PFS) rates using the method of Kaplan and Meier (25).

## Results

### Demographics

We identified 59 patients, who received first-line ipilimumab plus nivolumab therapy for metastatic cutaneous melanoma. These patients were treated between October 2013 to January 2025. Patients were treated and monitored in a consistent fashion by a single physician (WS). Patient characteristics are provided (**Supplementary Table 1**). Patients had a potential median follow-up of 52.5 ± 23.1 months (range 24.8-134.6 months). The median age was 61.3 years (range 20.3–95 years). Thirty-seven percent of the patients received a “standard-dose” regimen, while 63% received the alternate or “flipped-dose” regimen (21).

### Development of endocrinopathy

**Figure 1:**
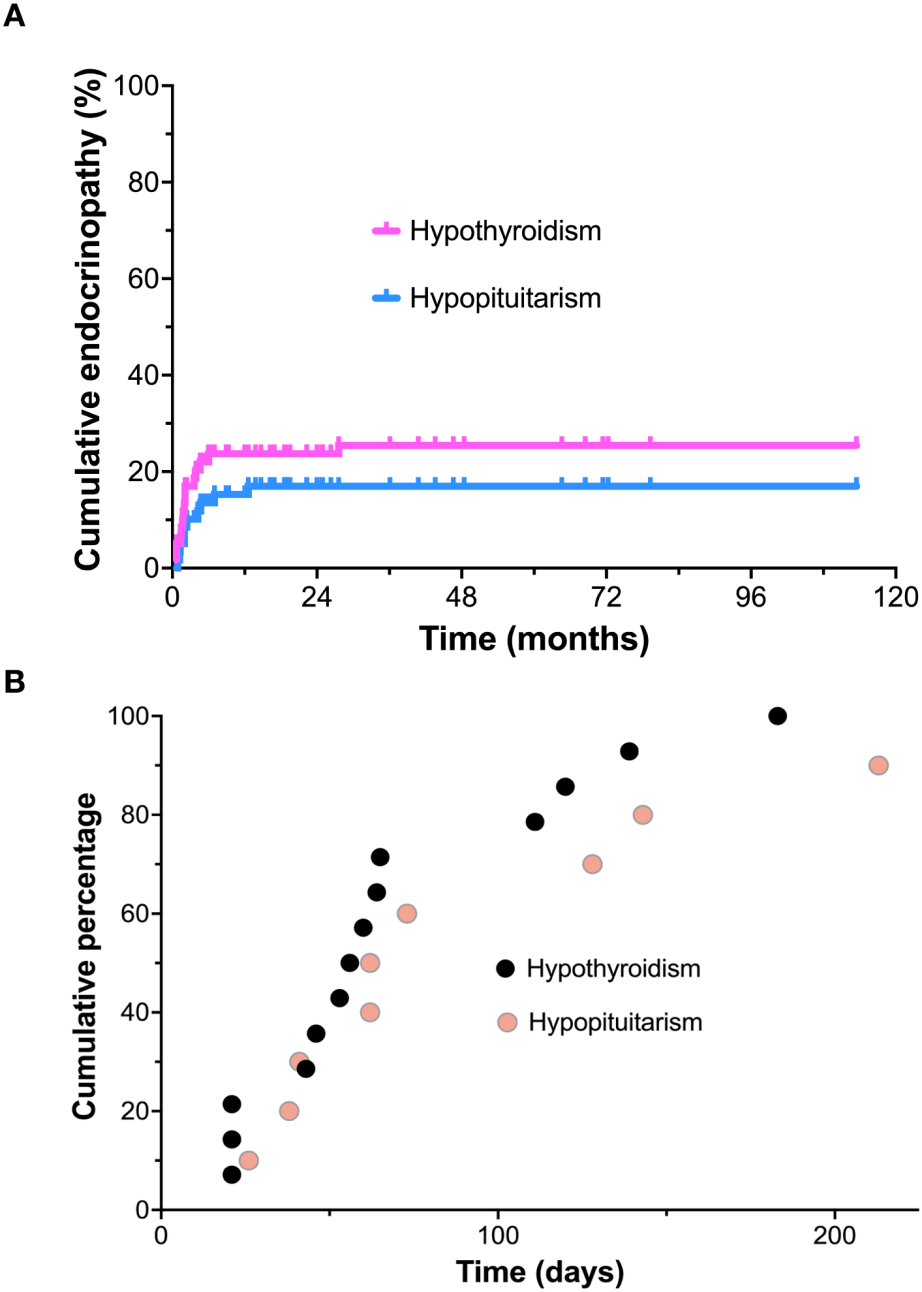
Time to onset of hypopituitarism or hypothyroidism from the initial dose of ipilimumab plus nivolumab. **(A)** Cumulative incidence of hypopituitarism and hypothyroidism; **(B)** Time to onset of hypopituitarism and hypothyroidism (days from start of therapy).

The earliest onset of endocrinopathy identified in a patient was 17 days after the first dose of ipilimumab plus nivolumab. A total of 26 of 59 (44.1%) of our patients treated with ipilimumab plus nivolumab developed biochemical evidence for either hypopituitarism or hypothyroidism, requiring pharmacologic replacement (**Figure 1A**). This included 18.7% with hypopituitarism associated with central hypothyroidism and secondary hypoadrenalism. In addition, 25.4% developed isolated hypothyroidism. We did not identify any patients who developed type 1 diabetes or primary adrenal insufficiency in this small patient series.

Seventeen patients of 59 total patients (28.8%) developed endocrinopathy within the first 90 days (during dual ICI therapy). The median time to onset of hypothyroidism was 58 ± 48.7 days, while the median time to onset for hypopituitarism was slightly more delayed, at 72.5 ± 107.3 days (**Figure 1B**).

Another 8 patients developed endocrinopathy during maintenance therapy with nivolumab monotherapy (10.3%). Only one patient was hospitalized due to symptomatic hypopituitarism, due to these early detection efforts and prompt endocrine replacement therapy. Delayed onset of endocrinopathy after the initial ICI treatment was a rare event in this sequentially monitored patient series. Only one of 26 patient developed delayed onset of hypothyroidism three months after elective treatment discontinuation. (1.7%). No patients developed endocrinopathy during longer follow-up, often spanning many years.

### Effect of endocrinopathy on clinical outcome of combination checkpoint inhibitor treatment

**Figure 2:**
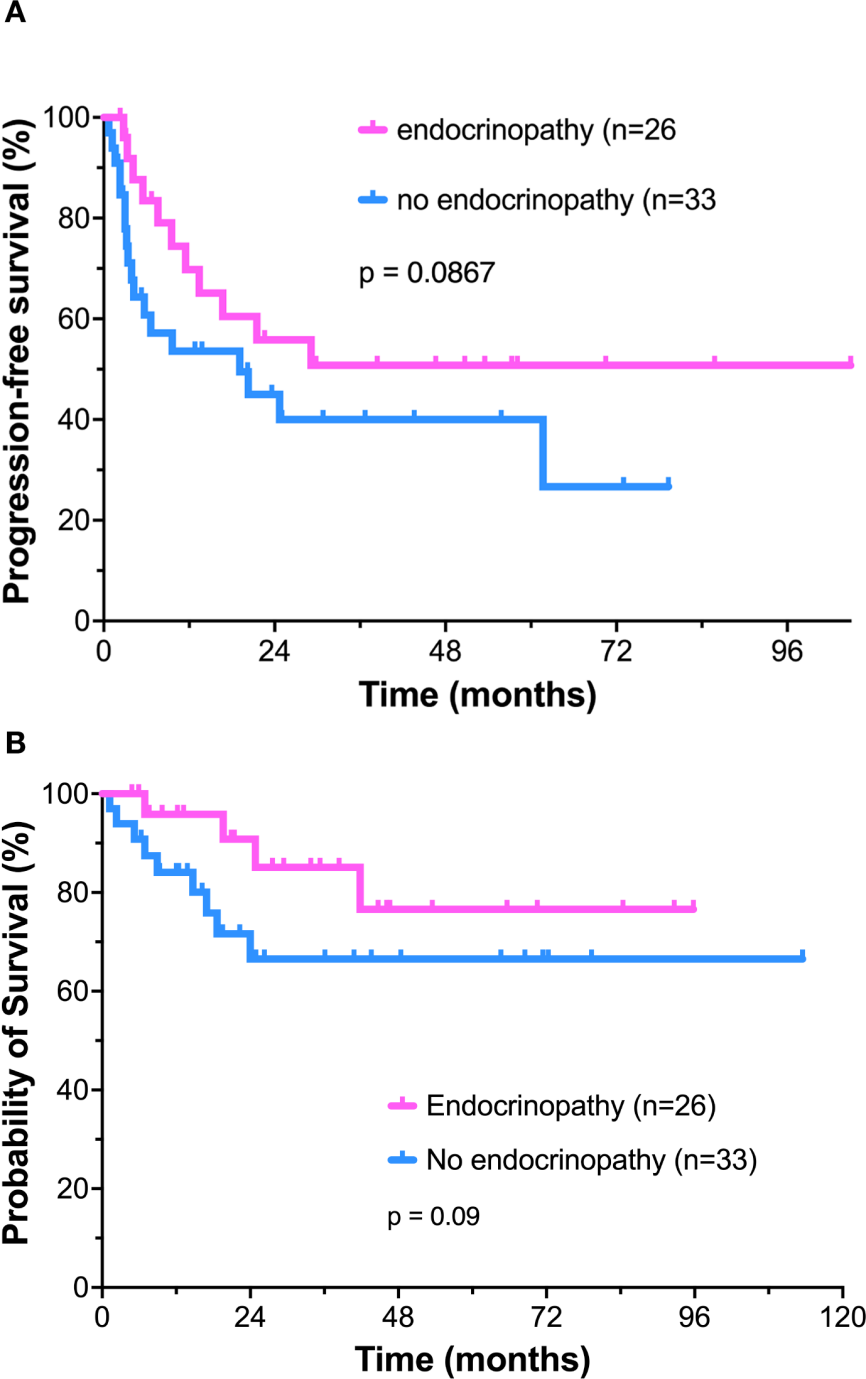
Comparison of clinical outcomes of patients who developed endocrinopathy versus those without. **(A)** Progression-free survival; **(B)** Overall survival.

It has been proposed that patients who develop immune-mediated toxicity have an increased likelihood of anticancer response. In our patient cohort, there was a trend toward improvement in PFS in patients who developed endocrinopathy compared to patients who did not experience this toxicity. Estimated 4-year PFS for these patients was 53.3% compared to 40.0% in patients without endocrine toxicity (**Figure 2A**)(p=.0867). Estimated overall survival was 76.6% at 4 years in patients who developed endocrinopathy, compared to 66.5% in those without (**Figure 2B**), however this also did not reach statistical significance (p=0.09).

**Figure 3:**
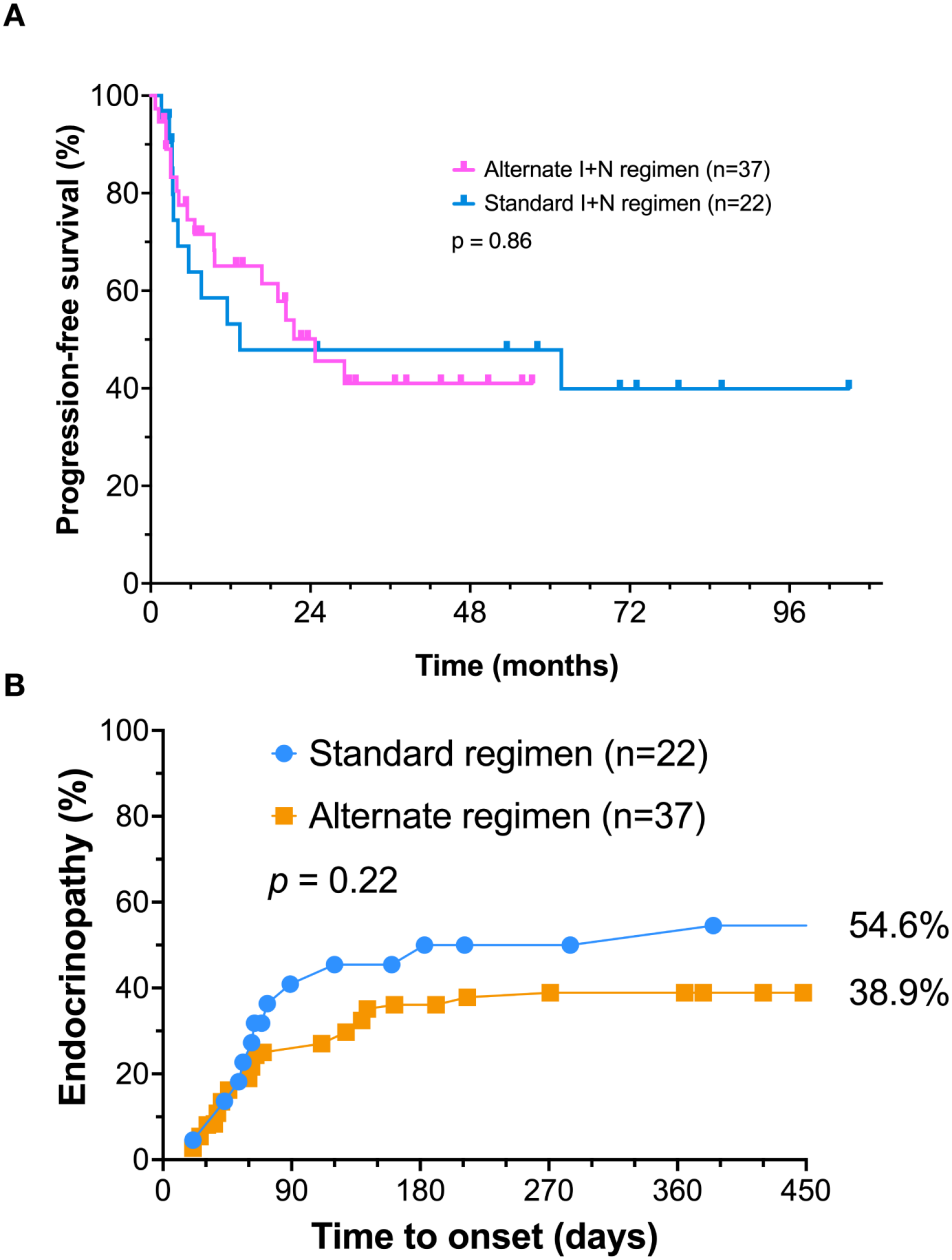
Exploratory analysis of the impact of the standard versus alternate ipilimumab plus nivolumab treatment regimen. **(A)** Progression-free survival; **(B)** Cumulative incidence of endocrinopathy over time.

In an exploratory analysis, the outcome of standard dose treatment and alternate dose treatment were also evaluated. There did not appear to be a difference in progression-free survival (p=0.86) between these two alternate regimens (**Figure 3A**). There was a trend toward an increased incidence of endocrinopathy with the standard I+N regimen (54.6%). However, the alternate regimen also resulted in a high (38.9%) incidence endocrinopathy (**Figure 3B**). This difference did not reach statistical significance (*p* = 0.22).

## Discussion

Combination immunotherapy with ipilimumab and nivolumab results in improved survival of patients with metastatic melanoma (26). Unfortunately, dual-ICI treatment is clinically challenging to administer, due to the significant risk of irAE. While most irAE are transient and can be controlled with immunosuppressive agents, there is a significant risk of longer-term endocrinopathies, such as hypothyroidism and hypopituitarism (27).

Interpretation of biochemical findings suggesting hypopituitarism requires consideration potential confounding disorders that may mimic secondary adrenal insufficiency. These include recent or ongoing systemic corticosteroid therapy, acute illness or physiologic stress, opioid use, and other medications that may suppress the hypothalamic–pituitary–adrenal axis (28, 29). In clinical practice, these factors may produce transient suppression of ACTH and cortisol levels, potentially complicating the interpretation of laboratory findings. In our retrospective analysis, medication history and clinical context were reviewed to exclude these disorders. All patients had normal baseline pituitary and thyroid function prior to the start of immunotherapy, strongly suggesting that the observed changes were treatment-related. In confusing cases, provocative testing such as ACTH stimulation testing or insulin tolerance testing could potentially be used to clarify the results.

The precise incidence of endocrinopathy, especially hypopituitarism, has been poorly defined. The variability of reports attempting to quantify the incidence of hypothyroidism and hypopituitarism is overviewed (**Table 1**). The extreme variability in incidence can be attributed to several factors. Many series included both patients treated with combination therapy, as well as checkpoint inhibitor monotherapy. In some clinical series, patients were evaluated only when overt clinical symptoms were detected. Since fatigue is a common side effect of all ICI treatment, significant delays in diagnosis and underestimates of the clinical incidence of endocrine abnormalities were likely in these studies. In addition, while thyroid function was routinely monitored during many clinical trials, pituitary function testing was less consistently required. This is evident, since many reported studies employed the term hypoadrenalism, when in fact changes in adrenal function were virtually always secondary to decreased anterior pituitary function (30).

**Table 1:** Published incidence of endocrine abnormalities following ICI therapy.

Endocrine abnormality	ICI MAb treatment	Published incidence	References
Hypothyroidism	CTLA-4	2.0-22.0%	(19, 31–37)
	PD-1	0.1-33.5%	(19, 31, 33–36, 38–43)
	CTLA-4+PD-1	1-41.9%	(19, 20, 31, 33, 35–38, 43, 44)
Hypopituitarism	CTLA-4	1.6-44.0%	(19, 30, 32–37, 45–48)
	PD-1	0.3-6.0%	(19, 30, 33–36, 41, 43, 47)
	CTLA-4+PD-1	0.5-44%	(19, 20, 30, 33, 35–37, 43, 44, 48)

Immune-related adverse events (irAEs) may occur because CTLA-4 and PD-1 pathways play a fundamental role in regulating autoimmunity, as well as in anti-tumor immunity (49, 50). CTLA-4 induction in T cells occurs early during the antigen response in draining lymph nodes, whereas PD1/PD-L1 pathway activation is believed to occur in peripheral tissues and within the tumor microenvironment (51). Thus, anti-CTLA-4 therapy can generate *de novo* reactive effector T cells directed against endocrine tissues, while anti-PD-1 therapy may activate pre-existing autoimmune T cells (52). Combination therapy with ipilimumab and nivolumab is, therefore, likely to facilitate activation of a broader spectrum of autoreactive T cells. The pathogenesis of endocrinopathy has also been hypothesized to relate to pre-existing low-grade autoimmunity to thyroid or pituitary tissue (53, 54). This may induce increased levels of PD-L1 expression in areas of thyroid and pituitary inflammation, as well as in pituitary adenomas (55–57).

In our current study, all patients were systematically monitored with biochemical endocrine testing during each treatment visit (every 3–4 weeks during active therapy). Due to this biochemical screening, we identified a higher-than-expected frequency of hypopituitarism in ipilimumab plus nivolumab treated patients. We identified hypopituitarism in 18.7%. Hypothyroidism occurred in 25.4% of patients. While routine monitoring of thyroid function during immunotherapy is customary, hypopituitarism is often overlooked as routine monitoring has not been recommended. Thus, hypopituitarism frequently presents with significant clinical symptoms. Due to our systematic biochemical monitoring protocol, we almost always detected this complication of immunotherapy in a presymptomatic state. Due to prompt hormone replacement, it was rare for our patients to develop overt clinical symptoms. Only one patient was hospitalized due to rapid onset of symptoms due to hypopituitarism. In general, this allowed treatment to be continued in an uninterrupted fashion. Recovery of endocrine function appeared to be rare despite lengthy follow-up after elective treatment discontinuation.

There have been efforts to decrease the substantial toxicity of I+N therapy. For example, an alternate treatment regimen, flipping the dose of ipilimumab and nivolumab has demonstrated similar clinical outcomes (21). This regimen substantially reduced the incidence of serious irAE from 48.3% to 33.3%. It should be noted that endocrinopathy was not considered a serious irAE in this study. An exploratory analysis of our patient data confirmed a similar PFS with both of these dosing regimens. The incidence of endocrinopathy with the standard dose regimen was 54.6%. However, the alternate dosing regimen still caused biochemical evidence for endocrinopathy in 38.9% of patients. We suspect that the standard regimen is likely to produce a higher rate of endocrinopathy, however this difference did not reach statistical significance, due to the small number of patients in our series.

Patients who developed endocrinopathy in our series had a trend toward both improved PFS and OS. This potential for improved treatment outcomes in patients that developed endocrinopathy has also been proposed by other investigators (47, 58–60). However, in our current study, neither PFS nor OS differences achieved statistical significance, most likely related to the relatively small number of patients.

In terms of timing of onset of endocrinopathy, most patients who developed endocrinopathy were identified during the initial 4 doses of combined ipilimumab plus nivolumab treatment (12 weeks). The median time to onset of hypothyroidism was 58 ± 48.7 days, while the median time to onset for hypopituitarism was 72.5 ± 107.3 days. Thus, hypopituitarism tended to occur in more delayed fashion (requiring more protracted monitoring). A small additional number of patients developed endocrinopathy during maintenance therapy with nivolumab. Only one patient developed delayed hypothyroidism that was identified 3 months following completion of immunotherapy and elective treatment discontinuation. No patients were found to have endocrinopathy more than 3 months after the completion of immunotherapy, despite lengthy follow-up. Thus, close monitoring of thyroid and pituitary function during dual ICI induction therapy and maintenance therapy seems mandatory. Our practice has been to monitor TSH, free-T4, cortisol and ACTH on each treatment visit. Monitoring should certainly continue during nivolumab maintenance therapy and perhaps for as long as 3–6 months following completion. The significance of our screening program was that very few patients became symptomatic or required hospitalization due to endocrine toxicity. Based on our observations, it would be reasonable to discontinue routine endocrine monitoring 6 months following completion of treatment, due to a very low risk of subsequent development of endocrinopathy. A that point, endocrine testing should only be performed in clinically symptomatic patients.

The strengths of this study include consistent monitoring for development of pre-symptomatic endocrinopathy and treatment of all patients in a consistent manner by a single oncologist. The limitations of the study include a relatively small sample size (59 patients). In addition, routine endocrine monitoring focused primarily on thyroid and adrenal axis function(TSH,FT4, ACTH, and cortisol), and other pituitary hormones such as LH/FSH, prolactin, or GH/IGF-1 were not systemically evaluated. Therefore, additional pituitary hormone abnormalities may have been under-recognized. While the study included all patients treated in our clinic with first line ipilimumab plus nivolumab, the study was retrospective in nature and requires further confirmation in prospective trials. A larger sample size is likely to have shown a stronger statistical relationship between the development of endocrinopathy and both OS, and PFS. It is also not clear from this retrospective analysis whether endocrinopathy would have eventually resolved in some patients following completion of therapy. Given the relatively small sample size and retrospective design of the study, definitive conclusions regarding optimal duration of endocrine monitoring cannot be made. While our observations suggest that most endocrinopathy occur during the treatment or shortly thereafter, larger prospective studies with longer follow-up are required to determine whether routine endocrine monitoring beyond 3–6 months after treatment completion is necessary. Thus, this study should be considered hypothesis generating.

## Conclusions

We evaluated the development of endocrinopathy as an immune-related adverse effect related to first-line ipilimumab plus nivolumab therapy in patients with metastatic melanoma. The onset of endocrinopathy most frequently occurred during the initial 4 doses of combination therapy (median onset < 60 days). The incidence of endocrinopathy following both the standard I+N regimen or the alternate treatment regimen was surprisingly high. Endocrinopathy usually presented as either primary hypothyroidism or hypopituitarism (including central hypothyroidism and secondary hypoadrenalism). These occurred in 25.4 and 18.7% of treated patients respectively. Early detection and intervention prevented hospitalizations and treatment delays. Biochemical evidence for hypopituitarism was detected at a higher-than-expected incidence. Based the high incidence of hypopituitarism we identified, routine endocrine screening is strongly recommended. It is likely that early detection and intervention before patients become symptomatic can decrease morbidity and hospitalizations. Delayed onset of endocrinopathy following ICI treatment discontinuation was quite rare in patients who achieved a remission (1.7%). The possibility that routine endocrine monitoring may not be necessary for more than 3–6 months after elective treatment discontinuation in responding patients will require confirmation in prospective trials. Patients who developed endocrinopathy tended toward improved OS and PFS. Thus, our data strongly suggests that routine monitoring of pituitary function should be incorporated during combination checkpoint inhibitor therapy.

## Data Availability

The original contributions presented in the study are included in the article/**Supplementary Material**. Further inquiries can be directed to the corresponding author.
