# Supplementary material for: Systematic monitoring identified a high incidence of hypopituitarism following combined ipilimumab plus nivolumab therapy for metastatic melanoma
Source: Front Endocrinol (Lausanne). 2026 May 8;17:1827644. doi: 10.3389/fendo.2026.1827644 (PMC13193898; doi:10.3389/fendo.2026.1827644)
Supplement: Supplementary file 1 [file Table1.docx]

**Supplemental Data:**

**Table 1 Patient demographics**

| **UPN** | **Age** | **Gender** | **Race** | **Driver Mutation** | **LDH (IU/ml)** | **Sites of Metastases** |
| --- | --- | --- | --- | --- | --- | --- |
| 1 | 69 | M | W | NRAS | 171 | Liver, Lung, LN |
| 2 | 57 | M | W | NRAS | 170 | Chest Wall, LN |
| 3 | 59 | M | W | NF-1 |  | Lung, Brain |
| 4 | 75 | F | W | NA | 213 | Lung |
| 5 | 95 | M | W | NA | 218 | LN |
| 6 | 68 | F | W | BRAF int rearrangement | 190 | LN, Bone |
| 7 | 45 | M | W | NF-1 | 281 | LN |
| 8 | 41 | F | W | BRAF V600E | 164 | LN |
| 9 | 59 | M | W | BRAF V600E | 654 | Lung, Bone, Gastric, LN |
| 10 | 75 | F | W | BRAF V600E | 284 | Lung, LN |
| 11 | 59 | M | W | NA | 168 | Adrenal, LN |
| 12 | 68 | M | W | BRAF V600K | 181 | Lung, Paraspinal, Bone, Breast |
| 13 | 46 | F | N/A | NA | 181 | Lung, SQ |
| 14 | 66 | M | W | NRAS | 159 | Lung, LN, Liver |
| 15 | 73 | F | W | QN | 202 | lung, LN |
| 16 | 67 | M | W | NA | 194 | SQ, LN |
| 17 | 56 | M | W | BRAF V600E | 215 | SQ, peripancreatic, bone |
| 18 | 79 | M | W | NF-1 | 215 | LN |
| 19 | 61 | M | W | NA | 196 | SQ |
| 20 | 73 | F | H | NF-1 | 231 | LN, Lung, Liver |
| 21 | 71 | F | W | NA | 219 | SQ, liver, lung, LN |
| 22 | 37 | M | W | NA | 206 | LN, SQ |
| 23 | 59 | F | W | NA | 153 | Lung, liver, LN |
| 24 | 70 | M | W | NRAS | 305 | Lung |
| 25 | 54 | M | H | QN | 213 | LN, Lung, Liver, Pancreas |
| 26 | 65 | M | W | QN | 165 | Bone, LN, Soft Tissue |
| 27 | 53 | M | W | BRAF V600K | 153 | SQ |
| 28 | 60 | M | W | NA | 193 | Lung |
| 29 | 70 | M | W | BRAF V600E | 683 | LN, Bone, Spinal meninges |
| 30 | 64 | F | W | QN | 305 | SQ |
| 31 | 48 | M | W | BRAF V600E | 184 | SQ |
| 32 | 48 | M | W | NF-1 | 151 | Lung |
| 33 | 76 | F | W | QN |  | locally advanced |
| 34 | 63 | F | W | BRAF V600E | 172 | GI, Lung |
| 35 | 49 | F | W | NA | 293 | LN, Pancreas, Abdomen, Lung |
| 36 | 87 | M | W | BRAF V600E | 359 | Liver, Bone, LN |
| 37 | 73 | F | W | NA | 214 | LN |
| 38 | 77 | M | W | NF-1 | 281 | Pancreas, Liver, Bone |
| 39 | 49 | M | W | NRAS | 191 | SQ, LN |
| 40 | 40 | M | W | NA | 167 | SQ, bone, LN |
| 41 | 20 | F | W | BRAF insertion | 193 | LN, Lung |
| 42 | 48 | M | W | BRAF V600E | 139 | Lung, LN, SQ |
| 43 | 73 | F | W | C-KIT | 154 | SQ, Bone, Lung |
| 44 | 75 | F | PI | HRAS | 181 | LN |
| 45 | 46 | M | W | BRAF V600E | 176 | SQ, Bone, Lung |
| 46 | 51 | F | W | BRAF V600K | 109 | Lung, LN |
| 47 | 81 | M | W | NA | 161 | Lung |
| 48 | 58 | M | H | KRAS | 187 | Liver, Bone |
| 49 | 63 | M | W | QN | 160 | SQ, LN, Liver |
| 50 | 46 | F | PI | NA | 138 | Lung, SQ, LN |
| 51 | 26 | M | W | BRAF V600E | 161 | LN, Adrenal, Bone |
| 52 | 43 | M | W | NA | 157 | LN |
| 53 | 44 | M | W | RAF-1 | 240 | LN, Liver, Bone |
| 54 | 66 | F | W | BRAF V600E | 196 | SQ, LN |
| 55 | 65 | M | W | NRAS | 184 | LN |
| 56 | 37 | M | W | BRAF V600E | 159 | SQ, LN |
| 57 | 72 | F | W | BRAF V600K | 169 | LN, Brain |
| 58 | 64 | M | W | BRAF V600K | 462 | Lung, SQ |
| 59 | 42 | M | H | NA | 275 | LN, Brain |

UPN, Unique Patient Number; M, Male; F, Female; W, White; H, Hispanic; PI, Pacific Islander; NA, not available; QN, quadruple negative genotype (no RAF, RAS, KIT or NF-1 mutation); LN, Lymph Node; SQ, subcutaneous.
