# Supplementary material for: Systematic monitoring identified a high incidence of hypopituitarism following combined ipilimumab plus nivolumab therapy for metastatic melanoma
Source: Front Endocrinol (Lausanne). 2026 May 8;17:1827644. doi: 10.3389/fendo.2026.1827644 (PMC13193898; doi:10.3389/fendo.2026.1827644)
Supplement: Supplementary file 2 [file Table2.docx]

**Supplemental Data:**

**Table 2 Patient outcomes**

| **UPN** | **I+N regimen** | **Potential f/u (months)** | **Pre-existing hypothyroidism** | **ICI induced**  **endocrinopathy** | **Endocrinopathy onset (days)** | **PFS**  **(mo)** | **OS**  **(mo)** | **Current Status** |
| --- | --- | --- | --- | --- | --- | --- | --- | --- |
| 1 | Alt | 26.0 | N | – |  | 2.3 | 18.6 | DOD |
| 2 | Std | 55.6 | N | T | 43 | 58.1 | 65.6 | NED |
| 3 | Std | 62.5 | N | – |  | 3.2 | 5.2 | DOD |
| 4 | Alt | 19.3 | N | – |  | 9.6 | 18.8 | NED |
| 5 | Alt | 19.1 | N | – |  | 1.2 | 1.2 | DOD |
| 6 | Alt | 38.4 | N | T | 139 | 16.7 | 19.6 | DOD |
| 7 | Alt | 11.6 | N | – |  | 5.3 | 5.3 | NED |
| 8 | Alt | 2.2 | N | P | 143 | 6.7 | 20.9 | NED |
| 9 | Alt | 45.4 | N | P | 26 | 29.1 | 41.8 | DOD |
| 10 | Alt | 16.6 | Y | – |  | 0.7 | 8.9 | DOD |
| 11 | Std | 66.2 | N | T | 56 | 4.1 | 6.9 | DOD |
| 12 | Std | 51.0 | N | P | 385 | 53.5 | 53.5 | NED |
| 13 | Std | 110.7 | N | – |  | 61.7 | 113.5 | NED |
| 14 | Alt | 18.5 | N | P | 38 | 5.5 | 33.8 | NED |
| 15 | Alt | 33.4 | N | – |  | 23.6 | 24.0 | D-other |
| 16 | Alt | 6.7 | N | T | 111 | 29.8 | 21.4 | NED |
| 17 | Alt | 28.5 | N | – |  | 12.8 | 16.2 | NED |
| 18 | Alt | 27.4 | N | – |  | 43.6 | 43.6 | NED |
| 19 | Alt | 11.5 | N | – |  | 13.8 | 13.8 | NED |
| 20 | Std | 0.8 | N | – |  | 1.6 | 6.9 | DOD |
| 21 | Std | 59.5 | Y | – |  | 73.0 | 72.3 | NED |
| 22 | Alt | 9.2 | N | – |  | 30.8 | 26.3 | NED |
| 23 | Std | 71.2 | Y | T |  | 13.4 | 27.6 | NED |
| 24 | Alt | 27.6 | N | T | 21 | 50.7 | 44.7 | NED |
| 25 | Alt | 15.0 | N | – |  | 3.0 | 14.7 | DOD |
| 26 | Alt | 12.2 | N | – |  | 3.9 | 12.4 | NED |
| 27 | Alt | 13.1 | N | P | 41 | 22.6 | 35.3 | NED |
| 28 | Std | 32.0 | N | – |  | 2.3 | 2.3 | NED |
| 29 | Std | 67.9 | N | – |  | 2.7 | 9.3 | NED |
| 30 | Std | 53.6 | N | T | 21 | 2.8 | 12.2 | NED |
| 31 | Alt | 33.6 | N | P | 128 | 57.3 | 29.4 | NED |
| 32 | Std | 52.9 | N | – |  | 3.4 | 68.5 | NED |
| 33 | Std | 58.8 | N | T | 21 | 11.5 | 13.2 | NED |
| 34 | Alt | 26.8 | N | P |  | 46.6 | 46.6 | NED |
| 35 | Alt | 25.6 | N | T | 46 | 21.5 | 24.8 | DOD |
| 36 | Alt | 28.5 | N | – |  | 2.0 | 2.3 | D-other |
| 37 | Std | 56.9 | N | – |  | 79.3 | 79.3 | NED |
| 38 | Alt | 32.0 | N | – |  | 55.8 | 48.4 | NED |
| 39 | Alt | 41.7 | N | – |  | 20.3 | 64.6 | NED |
| 40 | Alt | 15.5 | N | T | 65 | 7.6 | 7.6 | NED |
| 41 | Std | 69.5 | N | T | 53 | 3.1 | 4.8 | NED |
| 42 | Alt | 23.0 | N | P | 62 | 9.5 | 46.1 | NED |
| 43 | Alt | 13.8 | N | – |  | 6.6 | 16.9 | DOD |
| 44 | Alt | 26.2 | N | – |  | 4.2 | 6.3 | NED |
| 45 | Std | 81.3 | N | P | 73 | 3.3 | 95.8 | NED |
| 46 | Alt | 17.5 | N | – |  | 24.7 | 24.8 | NED |
| 47 | Std | 47.3 | N | T | 120 | 70.5 | 70.5 | NED |
| 48 | Alt | 30.4 | N | – |  | 2.3 | 12.0 | NED |
| 49 | Alt | 39.5 | N | – |  | 3.0 | 40.8 | NED |
| 50 | Alt | 9.7 | N | T | 60 | 2.3 | 5.9 | NED |
| 51 | Std | 52.2 | N | – |  | 5.7 | 71.4 | NED |
| 52 | Std | 62.0 | Y | T | 64 | 85.8 | 84.4 | NED |
| 53 | Alt | 15.5 | N | – |  | 36.7 | 36.1 | NED |
| 54 | Std | 27.4 | Y | – |  | 25.0 | 22.3 | NED |
| 55 | Alt | 48.1 | N | – |  | 19.1 | 24.9 | NED |
| 56 | Alt | 20.1 | N | – |  | 20.2 | 19.5 | NED |
| 57 | Alt | 17.5 | N | P | 213 | 38.4 | 38.4 | NED |
| 58 | Std | 81.1 | N | P | 62 | 104.9 | 92.7 | NED |
| 59 | Std | 50.9 | N | T | 183 | 7.6 | 9.7 | NED |

UPN, Unique Patient Number; Std, standard ipilimumab plus nivolumab regimen; Alt, alternate ipilimumab plus nivolumab regimen; Y, Yes; N, No; T, Hypothyroidism; P, Hypopituitarism; NED, No Evidence of Disease; AWD, Alive With Disease; DOD, Died Of Disease; D-other, died of non-melanoma causes.
